# Comprehensive Characterization of RNA Processing Factors in Gastric Cancer Identifies a Prognostic Signature for Predicting Clinical Outcomes and Therapeutic Responses

**DOI:** 10.3389/fimmu.2021.719628

**Published:** 2021-08-03

**Authors:** Shenghan Lou, Fanzheng Meng, Xin Yin, Yao Zhang, Bangling Han, Yingwei Xue

**Affiliations:** ^1^Department of Gastroenterological Surgery, Harbin Medical University Cancer Hospital, Harbin, China; ^2^Department of General Surgery, The First Affiliated Hospital of University of Science and Technology of China, Hefei, China

**Keywords:** RNA processing factors, alternative splicing event, drug response, prognostic model, immune heterogeneity, gastric cancer

## Abstract

RNA processing converts primary transcript RNA into mature RNA. Altered RNA processing drives tumor initiation and maintenance, and may generate novel therapeutic opportunities. However, the role of RNA processing factors in gastric cancer (GC) has not been clearly elucidated. This study presents a comprehensive analysis exploring the clinical, molecular, immune, and drug response features underlying the RNA processing factors in GC. This study included 1079 GC cases from The Cancer Genome Atlas (TCGA, training set), our hospital cohort, and two other external validation sets (GSE15459, GSE62254). We developed an RNA processing-related prognostic signature using Cox regression with the least absolute shrinkage and selection operator (LASSO) penalty. The prognostic value of the signature was evaluated using a multiple-method approach. The genetic variants, pathway activation, immune heterogeneity, drug response, and splicing features associated with the risk signature were explored using bioinformatics methods. Among the tested 819 RNA processing genes, we identified five distinct RNA processing patterns with specific clinical outcomes and biological features. A 10-gene RNA processing-related prognostic signature, involving *ZBTB7A*, *METTL2B*, *CACTIN*, *TRUB2*, *POLDIP3*, *TSEN54*, *SUGP1*, *RBMS1*, *TGFB1*, and *PWP2*, was further identified. The signature was a powerful and robust prognosis factor in both the training and validation datasets. Notably, it could stratify the survival of patients with GC in specific tumor-node-metastasis (TNM) classification subgroups. We constructed a composite prognostic nomogram to facilitate clinical practice by integrating this signature with other clinical variables (TNM stage, age). Patients with low-risk scores were characterized with good clinical outcomes, proliferation, and metabolism hallmarks. Conversely, poor clinical outcome, invasion, and metastasis hallmarks were enriched in the high-risk group. The RNA processing signature was also involved in tumor microenvironment reprogramming and regulating alternative splicing, causing different drug response features between the two risk groups. The low-risk subgroup was characterized by high genomic instability, high alternative splicing and might benefit from the immunotherapy. Our findings highlight the prognostic value of RNA processing factors for patients with GC and provide insights into the specific clinical and molecular features underlying the RNA processing-related signature, which may be important for patient management and targeting treatment.

## Introduction

Gastric cancer (GC) is the third leading cause of cancer-related mortality and the fifth most frequently diagnosed malignancy worldwide (1), with almost 1,000,000 estimated new cases and 800,000 deaths each year (1, 2). Due to the lack of early symptoms, most patients with GC are usually diagnosed at an advanced stage (3). Despite effective treatment, relapse and metastasis are common in advanced GC, causing a fairly low 5-year survival rate (<20%) (4). To date, the tumor-node-metastasis (TNM) staging system remains the gold standard for predicting prognosis and guiding GC treatment decisions (5). However, the high heterogeneity leads to different outcomes among patients with the same TNM stage and treatments (6). Therefore, it is imperative to investigate the in-depth molecular mechanisms involved in GC occurrence and development to identify novel prognostic biomarkers and potential therapeutic targets.

RNA processing, connecting genotype to phenotype, is a process that converts the primary transcript RNA into mature RNA (7). RNA processing regulates activities as diverse as tissue-specific gene expression, apoptosis, and maturation of the immune response, among many others (8). Altered RNA processing functionally drives tumor initiation and maintenance, and may generate novel therapeutic opportunities (9). Given that dysregulated expression of RNA processing factors can contribute to abnormalities in a series of RNA processing phases, such as mRNA transport, editing, and decay (9), systematic examination of the role of RNA processing factors in GC is necessary.

RNA processing factors also function in intron removal and regulate alternative splicing events (ASEs) of individual genes (10). Aberrant selective RNA processing, especially alternative splicing, could cause a series of consequences, from changing the stability to adding or deleting structural domains and modifying the interactive relationship between proteins (11). Recently, we demonstrated that aberrant ASEs play an essential role in GC occurrence and development (12, 13). However, to date, the relationship between the dysregulated RNA processing factors and the aberrant ASEs has not been clearly elucidated.

In the present study, we systematically explored the expression profile of RNA processing factors and their prognostic values in 1079 patients with GC. We used three different GC cohorts, including RNA sequencing (RNA-seq) data and microarray data, to construct and validate the RNA processing-related prognostic signature. We constructed a composite prognostic nomogram to facilitate clinical practice by integrating this RNA processing-related signature with age and tumor stage. Then, we analyzed the association between the signature and clinical outcomes, genetic variants, pathway activation, immune heterogeneity, and drug response features. Besides, we profiled the ASEs underlying GC stratified by this risk signature and identified the corresponding functions.

## Materials and Methods

### Gastric Cancer Dataset Source

We obtained 214 fresh frozen tumor specimens and clinical data from patients with GC who underwent gastrectomy as primary treatment at the Harbin Medical University (HMU) Cancer Hospital to construct the HMU-GC cohort. All samples were collected after written informed consent had been obtained from the patients. The study was approved by the HMU Cancer Hospital Institutional Review Board. RNA isolation, library construction, and mRNA sequencing were performed by Novogene (Beijing, China). The data were deposited in the Gene Expression Omnibus (GEO) repository (PRJNA718168).

We also systematically searched public gene expression data and complete clinical annotation in GEO and The Cancer Genome Atlas (TCGA) database. GC cohorts that: 1) had <150 patients; 2) lacked raw CEL files; 3) lacked basic clinical information (sex, age, TNM stage); or 4) lacked survival information were removed from further evaluation. Finally, four eligible GC cohorts, our HMU-GC cohort and three public datasets (GSE15459, GSE62254, TCGA-STAD), were included in the study for further analysis.

### Data Preprocessing

For microarray data from the GEO database, the raw CEL files were downloaded. To calculate absolute mRNA expression levels, we used the RMA (Robust Multi-array Average) method provided through the affy package to obtain background-adjusted, quantile-normalized, and probe-level data-summarized values for all probe sets (14, 15). For high-throughput sequencing data from the HMU-GC and TCGA-STAD datasets, raw read count values were transformed into transcripts per kilobase million (TPM) values, which are more similar to those generated from microarrays and are more comparable between samples (16). Batch effects from non-biological technical biases were corrected using the ComBat algorithm in the sva package (17).

The Affymetrix probe ID from the microarray data was annotated to gene symbols according to the GPL570 platform. For multiple probes that mapped to one gene, the mean expression value was considered. The Ensembl ID for mRNAs from high-throughput sequencing data was transformed to gene symbols *via* the biomaRt package (18). The mRNAs with TPM values of <1 in over 90% of samples were considered transcriptional noise and filtered out.

### Collection of RNA Processing Factors

RNA processing factors, defined as genes that participate in any process involved in the conversion of ≥1 primary RNA transcripts into ≥1 mature RNA molecules, were first collected from the gene ontology (GO) term (GO:0006396) in the AmiGO database (19). RNA processing factors with sufficiently reliable expression, shared among the eligible GC cohorts, were retained for further analyses.

### Unsupervised Clustering for RNA Processing Factors

Unsupervised clustering analysis was performed *via* hierarchical consensus clustering to identify the distinct RNA processing patterns based on the expression of RNA processing factors to classify patients for further analysis. The optimal number of clusters and their stability were determined by the consensus clustering algorithm. The above steps were performed using the ConsensusClusterPlus package, and 1000 repetitions were conducted to guarantee the stability of classification (20).

Gene set variation analysis (GSVA) was performed with the GSVA package (21), using the hallmark gene sets downloaded from MSigDB (22) to generate enrichment scores for each pathway per sample. Subsequently, we compared the GSVA enrichment score to explore the differences in biological functions and pathways among the distinct clusters. The overall survival (OS) of patients in the different RNA processing clusters was compared with Kaplan-Meier survival analysis with log-rank testing.

### Identification of the RNA Processing-Related Prognostic Signature

Univariate Cox proportional hazards regression analysis was first performed on the expression matrix of RNA processing factors to estimate the relationship between RNA processing factors and prognosis (OS) in the TCGA-STAD cohort. RNA processing factors with p-value < 0.1 were selected as the potential prognosis-related RNA processing factors.

As the discovery cohort, the TCGA-STAD cohort was randomized into two subsets based on 5-fold sampling to enhance the robustness of this prognostic signature. The training set included 4-fold GC samples, and the internal testing set included the remaining 1-fold GC samples. The least absolute shrinkage and selection operator (LASSO) penalty was performed in the discovery cohort to build an optimal prognostic signature with the minimum number of RNA processing factors. Ten-fold cross-validation was conducted to tune the optimal value of the penalty parameter λ, which yields the minimum partial likelihood deviance. Finally, a set of RNA processing factors, the RNA processing-related prognostic signature, and their non-zero coefficients were identified.

The risk score**for the signature was calculated for each sample based on the following formula:

$$\text{Risk Score}=\sum_{i=1}^{n}Coef_{i}\times E_{i}.,$$

where *Coef_i_* is the coefficient and *E_i_* is the normalized expression value of each selected gene by log2 and z-score transformations. Patients were dichotomized into high-risk and low-risk groups using the cohort-specific median risk score as the cut-off. The performance of risk groups determined by the risk score was assessed based on the restricted mean survival (RMS) time difference between the high-risk and low-risk groups (23). Kaplan-Meier curves were generated for survival rates, with difference detection based on log-rank testing.

### Development and Verification of a Composite RNA Processing–Clinical Prognostic Nomogram

Based on the multivariate analyses results, we integrated age, TNM stage, and the RNA processing-related prognostic signature to generate a composite prognostic model by applying a Cox proportional hazard regression in the TCGA-STAD cohort. The corresponding coefficients derived from the TCGA-STAD cohort were then used in the other two validation sets (HMU and GEO) for further validation. The prognostic value of the composite prognostic model was compared with the TNM staging system in terms of the concordance index (C-index), revealed by the RMS curve (24). The RMS represents the life expectancy at 60 months for patients with different risk scores. Finally, a nomogram was generated for model visualization and clinical application. The performance of the nomogram was evaluated by time-dependent receiver operator characteristic (ROC) analysis, calibration curve, and decision curve analysis (DCA) (25).

### Construction of Regulatory Network Between RNA Processing Factors and ASEs

The corresponding alternative RNA splicing data of the TCGA-STAD cohort were downloaded from the TCGA SpliceSeq database (26). Splicing events in the dataset were divided into seven categories: exon skip (ES), retained intron (RI), alternate promoter (AP), alternate terminator (AT), alternate donor site (AD), alternate acceptor site (AA), and mutually exclusive exons (ME). To generate a reliable set of ASEs, we implemented a series of stringent filters, which included “percentage of samples with PSI value ≥ 75%” and “average PSI value ≥ 0.05”. Only ASEs meeting the above criteria were included for further analysis. Each splicing event was quantified by the percent spliced in (PSI) value (27), representing the ratio of included transcript reads in the total transcript reads.

To investigate the potential functions of RNA splicing, we performed enrichment analysis for all differential spliced genes in GC samples with lower risk (first quartile) and higher risk (fourth quartile) scores. These differential spliced genes were mapped to the Search Tool for the Retrieval of Interacting Genes/Proteins (STRING) database to observe the protein–protein interaction relationship (28). The protein interaction network was constructed to explore the potential impact of RNA splicing on protein-protein interactions in GC.

The potential association of the differential PSI values of ASEs between GC samples with lower and higher risk scores were predicted using RNA processing factors with significant expression levels. We calculated the Pearson’s correlation for each RNA processing factor-ASE pair. The RNA processing factor-ASE pair with absolute correlation coefficients > 0.5 and Benjamini-Hochberg adjusted p-value < 0.05 were considered significant. The potential regulatory network was visualized with Cytoscape (29).

### Immunohistochemical Analysis

Protein expression data were obtained from the Human Protein Atlas (HPA) database, the largest and most comprehensive database for evaluating protein distribution in human tissues (30). The protein expression of the selected RNA processing factors in normal and GC tissues was determined using the immunohistochemical staining images.

### Bioinformatics Analyses

GO and Kyoto Encyclopedia of Genes and Genomes (KEGG) pathway enrichment analyses were utilized for gene set functional annotation. The functional enrichment of risk score-associated genes was investigated in gene set enrichment analysis (GSEA) using the clusterProfiler package (31, 32). We also performed GSVA to determine the functional differences between the risk groups. The mutation landscape was created with the maftools package with the initial removal of 100 FLAGS (frequently mutated genes) (33, 34). The presence of infiltrating stromal and immune cells in tumors was estimated with the estimate package (35). The population abundance of tissue-infiltrating immune and stromal cell populations was assessed with the MCPcounter package (36).

The gene module associated with the RNA processing-related prognostic signature was identified using weighted correlation network analysis (WGCNA) according to the protocol and recommendations of the WGCNA package (37). A scale-free topology fitting index (R^2^) > 0.85 was set as the threshold to construct the weighted gene co-expression network. A minimum cluster size of 30 and a merge threshold function of 0.25 were chosen as the thresholds for identifying co-expressed gene modules. A biweight midcorrelation coefficient (r) ≥ 0.3 and p-value < 0.05 were set as the thresholds for determining gene modules associated with the prognostic signature.

Based on three public drug sensitivity databases, GDSC (Genomics of Drug Sensitivity in Cancer) (38), CTRP (Cancer Therapeutics Response Portal) (39), and PRISM (40), the pRRophetic package was applied for predicting chemotherapeutic response by using ridge regression to estimate the area under the dose–response curve (AUC) value for each sample (41, 42). The prediction accuracy was evaluated by 10-fold cross-validation based on each training set. Lower AUC values indicated increased sensitivity to treatment. Seven common chemotherapeutic agents (5-fluorouracil, cisplatin, oxaliplatin, capecitabine, paclitaxel, docetaxel, irinotecan) were selected for predicting the chemotherapeutic response (43). Furthermore, we predicted the relationship between the RNA processing-related prognostic signature and immunotherapy response using the Tumor Immune Dysfunction and Exclusion (TIDE) web tool (http://tide.dfci.harvard.edu/) (44). Patients with higher TIDE scores have a higher chance of antitumor immune escape, thereby exhibiting a lower immunotherapy response rate.

### Statistical Analyses

All statistical tests were performed with R statistical software (v4.0.2) using Mann-Whitney testing for continuous data and Fisher’s exact testing for categorical data. Correlation between two continuous variables was measured by Pearson’s correlation coefficient. The hazard ratio (HR) and 95% confidence intervals (CI) were estimated by a Cox regression model using the survival package. Survival analysis was carried out using Kaplan–Meier methods. The statistical significance of differences was determined using log-rank testing. The RMS curve and RMS time difference were estimated with the survRM2 package. The time-dependent AUC was computed using the timeROC package. The C-index was compared with the compareC packages. For all statistical analyses, a two-tailed p-value < 0.05 was considered significant.

## Results

### Overview of RNA Processing Factors in GC

A total of 1079 patients diagnosed with GC from four independent datasets (GSE15459, GSE62254, HMU-GC, TCGA-STAD) were ultimately included in this study. First, 929 genes, annotated as RNA processing factors in the GO term (GO:0006396), were acquired from the AmiGO database (**Supplementary Table 1**). After low-expression genes had been filtered out, 819 genes were present in all datasets (**Supplementary Table 2**). The entire workflow of this study, including the filtration of RNA processing factors, development and validation of a prognostic signature, the construction of a composite processing-clinical prognostic nomogram and, the analyses of signature-associated alteration of the ASEs and RNA expression profiles, are delineated in **Supplementary Figure 1**.

### Identification of the Five Distinct RNA Processing Patterns

**Figure 1:**
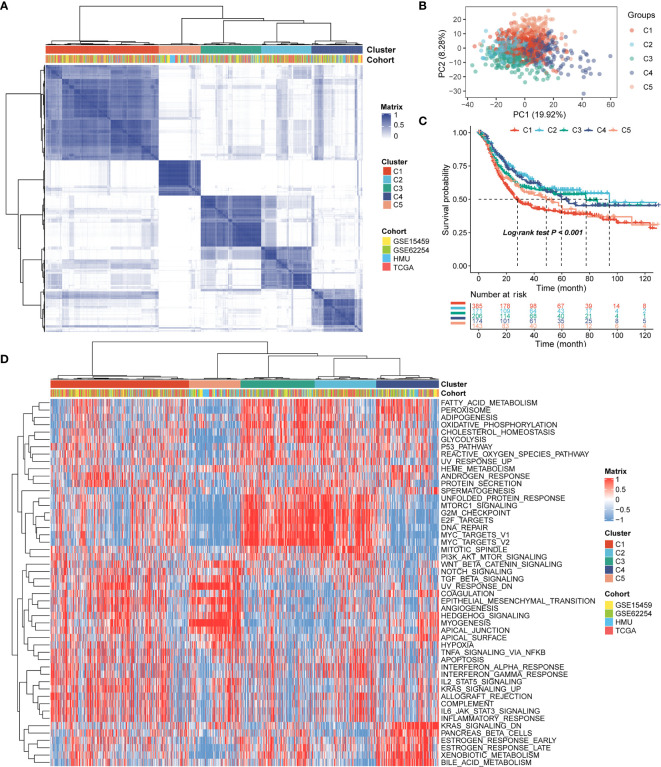
Identification of the five distinct RNA processing patterns. **(A)** Heatmap showing consensus clustering analysis for the five defined RNA processing patterns. **(B)** Scatter plots showing principal component analysis (PCA) of the five distinct RNA processing patterns. **(C)** Kaplan–Meier survival analysis of OS for patients with the five distinct RNA processing patterns. **(D)** Heatmap showing GSVA scores of the hallmark gene sets for the five defined RNA processing patterns.

Patients with qualitatively different RNA processing patterns were classified using a meta-cohort (GSE15459, GSE62254, HMU-STAD, TCGA-STAD). Five distinct patterns were eventually identified using unsupervised hierarchical clustering (**Figures 1A, B**): 385 cases in cluster 1, 171 cases in cluster 2, 206 cases in cluster 3, 174 cases in cluster 4, and 143 cases in cluster 5. Prognostic analysis of the five main RNA processing subtypes showed significant survival differences (log-rank test, p < 0.01; **Figure 1C**). Patients in clusters 2 and 3 had better prognosis than those in clusters 1 and 5 (**Figure 1C**).

We performed GSVA to explore the biological processes among these distinct RNA processing patterns. These five RNA processing subtypes showed significant enrichment of specific biological processes (**Figure 1D**). Clusters 2 and 3, correlated with good prognosis, were markedly enriched in the proliferation-specific pathways, such as the activation of the G2M checkpoint, E2F targets, and MYC targets pathway. Cluster 4, characterized by moderate prognosis, represented enriched pathways associated with metabolism activation, including the xenobiotic metabolism, bile acid metabolism, and estrogen response pathways. Clusters 1 and 5, associated with poor prognosis, were prominently related to stromal activation pathways, involving the epithelial–mesenchymal transition (EMT), transforming growth factor (TGF)-beta, and angiogenesis pathways. All these analyses suggest that RNA processing factors play an important role in GC occurrence and progression.

### Identification of the RNA Processing-Related Prognostic Signature

**Figure 2:**
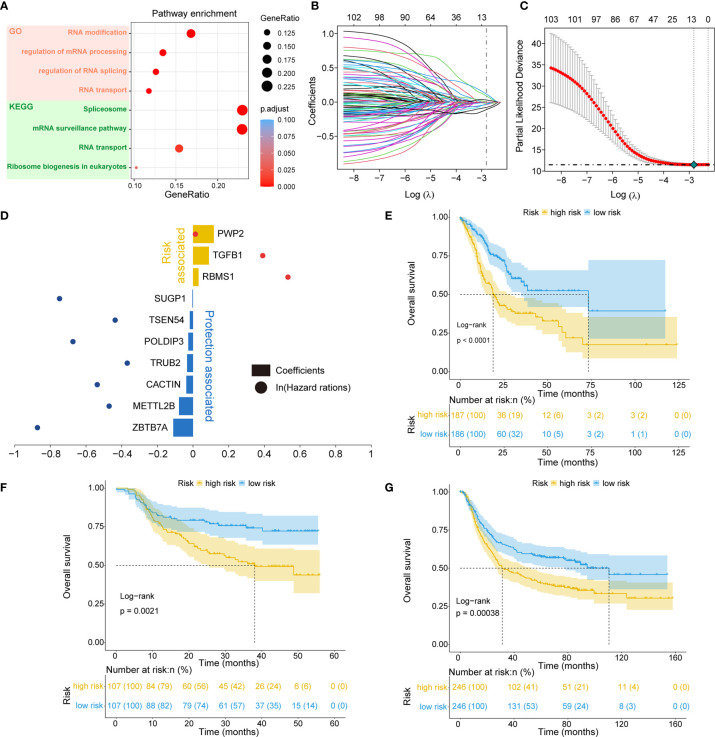
Identification of the RNA processing-related prognostic signature. **(A)** Scatter plots showing functional enrichment analyses for the 105 OS-related RNA processing factors. **(B, C)** LASSO regression analysis of the 105 OS-related RNA processing factors. **(D)** The 10 genes included in the signature. Corresponding coefficients and HRs are depicted by horizontal bars and dots, respectively. **(E–G)** Kaplan–Meier OS curves with difference detection by log-rank test for patients from the training and validation datasets.

Of the 819 RNA processing factors, 105 were associated with OS (**Supplementary Table 3**). Among these 105 factors, 51 factors (HR >1) were considered risk-associated, while the remaining 54 factors (HR <1) were considered protection-associated. We performed KEGG and GO functional enrichment analyses to study the more specific biological functions of these prognosis-related RNA processing factors. The results indicated that these factors were correlated with such key biological functions as RNA modification, regulation of RNA splicing, RNA transport, and spliceosome (**Figure 2A**).

To stratify the clinical outcomes of patients with the RNA processing factors readily and efficiently, we applied the LASSO Cox regression algorithm to the 105 factors in the TCGA training set. A total of 10 factors with non-zero coefficients were identified (**Figures 2B, C**). These LASSO-selected features were used to build the RNA processing-related signature (**Figure 2D**). The corresponding risk scores were computed for both the training and the validation datasets, according to the following formula:

Risk Score = −0.111×ZBTB7A−0.078×METTL2B−0.037×CACTIN−0.033×TRUB2−0.027×POLDIP3−0.018×TSEN54−0.003×SUGP1+0.031×RBMS1+0.089×TGFB1+0.116×PWP2

We divided patients in all three datasets into high-risk and low-risk groups using their respective median risk score as the cutoff. Kaplan-Meier survival analysis determined that patients with low-risk scores had significantly longer OS than those with high-risk scores (TCGA training set: p < 0.001, HR = 0.455, 95% CI: 0.324-0.638; HMU validation set: p = 0.002, HR = 0.487, 95% CI: 0.304-0.778; GEO validation set: p < 0.001, HR = 0.633, 95% CI: 0.491-0.817; **Figures 2E–G**). Significant RMS time differences were also observed between the low-risk and high-risk groups at different time points; the RMS time differences increased as the follow-up duration was extended (**Table 1**). For example, the RMST differences between the two groups were 1 (TCGA), 4 (HMU), and 0 (GEO) months for OS at the first year of follow-up, which reached 11 (TCGA), 9 (HMU), and 7 (GEO) months at the fifth year.

**Table 1:** RMS time (RMST) between the two risk groups at different time points.

Dataset	Time point	RMST^a^		RMST difference^b^	p-value
		Low risk (95% CI)	High risk (95% CI)		
**TCGA cohort (n = 373)**	**12 months**	11.236 (10.881, 11.590)	10.206 (9.722, 10.69)	1.030 (0.430, 1.629)	**<0.001**
	**36 months**	28.138 (26.304, 29.972)	21.273 (19.167, 23.378)	6.865 (4.073, 9.658)	**<0.001**
	**60 months**	40.810 (36.656, 44.964)	29.399 (25.410, 33.387)	11.411 (5.652, 17.170)	**<0.001**
**HMU cohort (n = 214)**	**12 months**	11.046 (10.594, 11.498)	11.072 (10.700, 11.444)	-0.026 (-0.612, 0.559)	0.93
	**36 months**	29.787 (27.582, 31.992)	25.915 (23.592, 28.237)	3.872 (0.670, 7.075)	**0.018**
	**60 months**	43.591 (39.832, 47.350)	34.980 (30.935, 39.025)	8.611 (3.089, 14.133)	**0.002**
**GEO cohort (n = 492)**	**12 months**	11.242 (10.965, 11.519)	11.115 (10.852, 11.377)	0.127 (-0.255, 0.509)	0.514
	**36 months**	28.479 (27.011, 29.948)	25.578 (24.070, 27.085)	2.901 (0.797, 5.006)	**0.007**
	**60 months**	42.934 (40.076, 45.792)	36.224 (33.359, 39.089)	6.710 (2.663, 10.757)	**0.001**

a RMST, months.

b RMST difference = RMST_low risk_ – RMST_high risk._ The bold value means the outcome is statistically significant.

We performed univariate and multivariate Cox regression analyses in the training and validation datasets to investigate the prognostic value of the RNA processing-related signature. The signature was the only prognostic factor in all three datasets (univariate cox analysis: p < 0.05; **Table 2**). After adjusting for other prognostic factors (age and TNM stage), the signature remained a significant independent prognostic factor in the HMU and TCGA cohorts (**Table 2**). Furthermore, we performed subgroup analyses according to age, sex, and TNM stage to explore the interaction effect between the signature and clinical characteristics. Subgroup analyses showed no statistically significant tests of interaction (**Table 3**), suggesting the robustness of this signature for different clinical features.

**Table 2:** Univariate and multivariate Cox analyses of the RNA processing-related signature.

Dataset	Factor	Univariate		Multivariate	
		HR (95% CI)	p*-*value	HR (95% CI)	p*-*value
**TCGA cohort** **(n = 373)**	**Risk score (increasing values)**	9.280 (4.746, 18.148)	**<0.001**	9.918 (4.926, 19.968)	**<0.001**
	**Age** **(increasing years)**	1.021 (1.005, 1.037)	**0.012**	1.027 (1.010, 1.045)	**0.002**
	**Sex** **(male vs. female)**	1.333 (0.936, 1.899)	0.112		
	**TNM stage** **(III + IV vs. I + II)**	1.891 (1.325, 2.698)	**<0.001**	2.101 (1.464, 3.015)	**<0.001**
**HMU cohort** **(n = 214)**	**Risk score (increasing values)**	2.819 (1.041, 7.637)	**0.041**	2.819 (1.041, 7.637)	**0.041**
	**Age** **(increasing years)**	1.009 (0.992, 1.027)	0.284		
	**Sex** **(male vs. female)**	0.984 (0.615, 1.574)	0.947		
	**TNM stage** **(III + IV vs. I + II)**	1.165 (0.710, 1.911)	0.545		
**GEO cohort** **(n = 492)**	**Risk score (increasing values)**	3.601 (2.002, 6.474)	**<0.001**	1.632 (0.879, 3.029)	0.121
	**Age** **(increasing years)**	1.006 (0.995, 1.018)	0.254		
	**Sex** **(male vs. female)**	1.056 (0.810, 1.377)	0.686		
	**TNM stage** **(III + IV vs. I + II)**	4.245 (3.097, 5.817)	**<0.001**	3.983 (2.878, 5.511)	**<0.001**

The bold value means the outcome is statistically significant.

**Table 3:** Subgroup analysis of the RNA processing-related signature.

Data set	Factor	Subgroup analysis			p-value for interaction
		Samples	HR (95% CI)	p-value	
**TCGA cohort** **(n = 373)**	**Sex**				
	Female	133.000	12.071 (3.737, 38.996)	**<0.001**	0.651
	Male	240.000	8.271 (3.611, 18.945)	**<0.001**	
	**Age**				
	≤60	120.000	18.021 (4.949, 65.616)	**<0.001**	0.268
	> 60	249.000	7.543 (3.498, 16.267)	**<0.001**	
	**Stage**				
	Early (I and II)	164.000	7.482 (2.349, 23.831)	**0.001**	0.660
	Advanced (III and IV)	186.000	11.097 (4.527, 27.202)	**<0.001**	
**HMU cohort** **(n = 214)**	**Sex**				
	Female	77.000	2.162 (0.315, 14.849)	0.433	0.666
	Male	136.000	3.372 (1.037, 10.971)	**0.043**	
	**Age**				
	≤60	118.000	2.369 (0.563, 9.977)	0.240	0.691
	> 60	96.000	3.509 (0.883, 13.943)	0.075	
	**Stage**				
	Early (I and II)	67.000	1.243 (0.213, 7.264)	0.809	0.260
	Advanced (III and IV)	147.000	4.149 (1.234, 13.947)	**0.021**	
**GEO cohort** **(n = 492)**	**Sex**				
	Female	168.000	6.633 (2.354, 18.691)	**<0.001**	0.113
	Male	324.000	2.651 (1.290, 5.447)	**0.008**	
	**Age**				
	≤60	178.000	8.792 (3.039, 25.438)	**<0.001**	0.065
	> 60	314.000	2.583 (1.260, 5.291)	**0.010**	
	**Stage**				
	Early (I and II)	187.000	2.796 (0.784, 9.976)	0.113	0.312
	Advanced (III and IV)	305.000	1.331 (0.654, 2.708)	0.431	

The bold value means the outcome is statistically significant.

### Identification of the Composite Prognostic Nomogram

**Figure 3:**
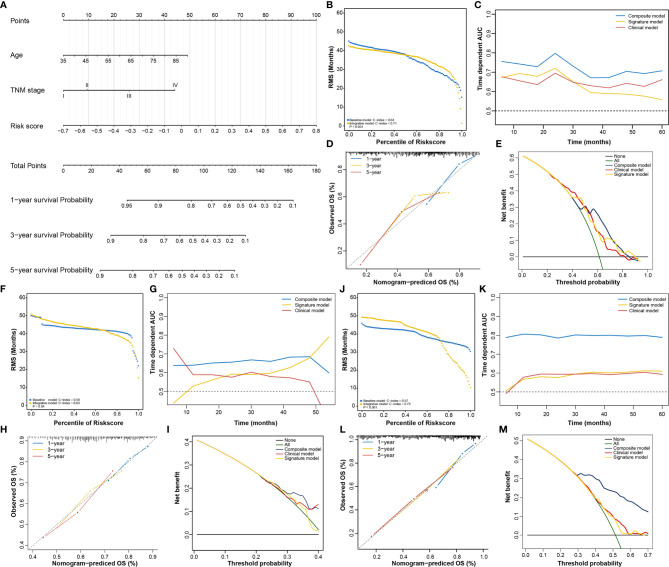
Identification of the composite prognostic nomogram. **(A)** Composite nomogram prediction of 1-year, 3-year, and 5-year OS. **(B, F, J)** RMS curves for the composite prognostic nomogram and clinical model in the training and validation datasets. **(C, G, K)** Time-dependent ROC curves for the nomogram, signature model, and clinical model at different time points in the training and validation datasets. **(D, H, L)** Calibration curves of observed and predicted probabilities for the nomogram in the training and validation datasets. **(E, I, M)** DCA curves for the nomogram in the training and validation datasets.

In addition to the RNA processing-related signature, clinical characteristics, including age and TNM stage, might also be independent prognostic factors, suggesting their complementary value (**Table 2**). We integrated the signature with these significant clinical variables to further improve the prognostic accuracy, using the coefficients generated from the multivariate Cox regression model in the discovery cohort (TCGA cohort) and derived a composite prognostic model. A nomogram was then established for model visualization and clinical application (**Figure 3A**). The composite nomogram achieved significant improvement for assessing survival relative to the clinical model involving age and TNM stage (**Figure 3B**). The composite nomogram also performed better than the RNA processing-related signature and the clinical model for predicting GC prognosis (**Figure 3C**). The calibration curve detected an optimal prediction between the nomogram prediction and actual observations (**Figure 3D**).

Finally, we compared the clinical net benefit of the composite nomogram with that of the other two models through DCA curves. The composite nomogram demonstrated a larger net benefit than the RNA processing-related signature and basic clinical model within most of the above threshold probabilities (**Figure 3E**), indicating that the nomogram had the best clinical utility for predicting prognosis in patients with GC. All these findings were verified in the HMU (**Figures 3F–I**) and GEO validation datasets (**Figures 3J–M**), suggesting the reliability and stability of our composite nomogram.

### Function Analysis of Genes Correlated With the RNA Processing-Related Prognostic Signature

**Figure 4:**
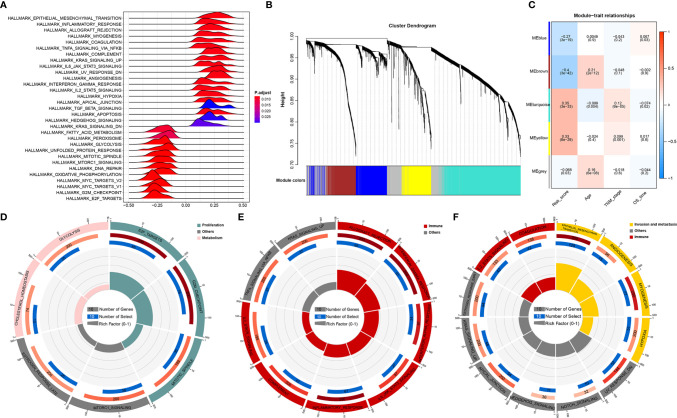
Function analysis of genes correlated with the RNA processing-related prognostic signature. **(A)** GSEA of the hallmark gene sets for risk scores based on pre-ranked Pearson’s correlation coefficients of risk score-associated mRNAs. **(B)** Clustering dendrogram of the top 5000 mRNAs with dissimilarity based on the topological overlap together with assigned module colors. **(C)** Module–trait relationships. Each row shows a module eigengene; each column corresponds to a clinical trait. Each cell contains the corresponding correlation (upper number) and p-value (lower number). **(D–F)** Functional enrichment analysis of the hallmark gene sets for the brown **(D)**, yellow **(E)**, and turquoise **(F)** modules.

Given that RNA processing factors are the main factors controlling the life cycle of RNAs in eukaryotes, we subsequently evaluated the RNA expression profile influenced by the RNA processing-related prognostic signature. In this case, we correlated the signature risk score with all robustly expressed mRNAs, generating a pre-ranked list sorted by the Pearson correlation coefficient, and further performed GSEA. The results indicated that invasion, metastasis, and immune hallmarks, such as EMT, myogenesis, angiogenesis, hypoxia, inflammatory response, interferon-gamma response, and complement, were significantly enriched in GC samples with higher risk scores. In contrast, proliferation and metabolism hallmarks, such as G2M checkpoint, MYC targets, oxidative phosphorylation, fatty acid metabolism, and glycolysis, were significantly enriched in GC samples with lower risk scores (**Figure 4A**).

Furthermore, we used WGCNA to obtain the signature-related modules according to the approximate scale-free features. The top 5000 most variant genes, measured by the median absolute deviation (MAD), were selected for the WGCNA. We chose nine as the optimal soft threshold power to calculate the adjacency matrix, which was the lowest threshold to enable the scale-free R^2^ to reach 0.85 (**Supplementary Figure 2**). We constructed a cluster dendrogram with the adjacency matrix; five color modules (blue, brown, turquoise, yellow, grey) were identified (**Figure 4B**). Genes that could not be included in any module were placed in the grey module and removed for the downstream analysis.

Next, we correlated the eigengene of the selected traits and modules to evaluate the module–trait relationships. Three modules (brown, turquoise, yellow) were highly significantly associated with the signature risk score (|R| > 0.3). The yellow and turquoise modules were positively correlated with the signature risk score. The brown module was negatively correlated with the signature risk score (**Figure 4C**). All modules also showed significant correlations between gene significance and module membership (**Supplementary Figure 3**), implying that the genes in these modules might play an essential biological role associated with the RNA processing-related prognostic signature.

We then performed functional enrichment analysis of the genes in each module to explore the biological functions of the signature-related modules. Consistent with the GSEA results, genes in the brown module were significantly enriched in the proliferation- and metabolism-related pathways (**Figure 4D**). For yellow module genes, the top enriched terms were allograft rejection, interferon-gamma response, and inflammatory response, suggesting that the yellow module is involved in the immune response (**Figure 4E**). Genes in the turquoise module were associated with the development of malignant phenotypes, focusing on invasion and metastasis processes (**Figure 4F**). These findings imply that the RNA processing-related prognostic signature reflects the expression alterations of genes involved in multiple vital hallmarks (invasion, metastasis, proliferation, metabolism, immune response) in GC.

### Expression and Clinical Features Underlying the RNA Processing-Related Prognostic Signature

**Figure 5:**
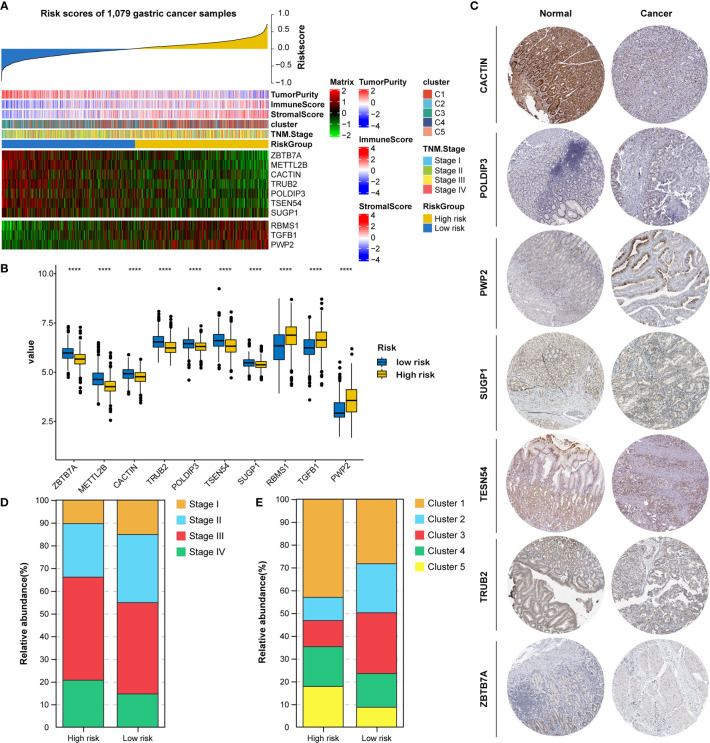
Identification of expression and clinical features underlying the RNA processing-related prognostic signature. **(A)** Heatmap showing the expression patterns of 10 prognosis-associated RNA processing factors for the entire 1079-sample GC set sorted by the signature risk score in ascending order. **(B)** Differential expression of the 10 prognosis-associated RNA processing factors between the low-risk and high-risk groups. P values were obtained by the Mann-Whitney test. The asterisks represented the statistical P-value (****P < 0.0001). **(C)** Histogram showing the distribution of TNM stage between the low-risk and high-risk groups. **(D)** Histogram showing the distribution of the five distinct RNA processing patterns between the low-risk and high-risk groups. **(E)** Immunohistochemical analysis of the protein expression of the 7 prognosis-associated RNA processing factors in the HPA database.

All 1079 GC samples were pooled to explore the expression and clinical features of the RNA processing-related prognostic signature. All 10 LASSO-selected factors were significantly differentially expressed between the two risk groups (**Figures 5A, B**). Risk-associated genes showed higher expression levels in patients with high risk scores. In comparison, protection-associated genes showed higher expression levels in those with low risk scores (**Figures 5A, B**). Moreover, the immunohistochemical analysis *via* the HPA determined that most protection-associated genes showed lower protein expression levels in GC samples than in adjacent normal tissues; the protein products of the risk-associated genes showed an opposite trend (**Figure 5C**).

Moreover, we found that advanced tumor stage (stage III and IV) was significantly enriched in the high-risk group (p < 0.001; **Figures 5A, D**). A higher percentage of clusters 1 and 5, featuring poor prognosis and stromal activation, was enriched in the high-risk group (p < 0.001; **Figures 5A, E**). These results suggest that the identified RNA processing factors might be involved in GC occurrence and development and could serve as potential therapeutic targets.

### Genetic Variants, Pathway Activation, and Immune Heterogeneity Underlying the RNA Processing-Related Prognostic Signature

**Figure 6:**
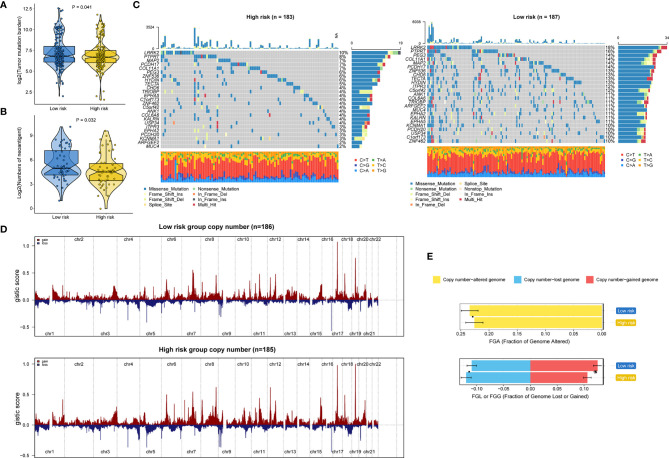
Identification of genetic variants underlying the RNA processing-related prognostic signature. **(A)** Violin plot for the TMB between the low-risk and high-risk groups. **(B)** Violin plot for the number of neoantigens between the lower-risk and higher-risk groups. **(C)** Waterfall plot of the top 25 mutant genes in the low-risk and high-risk groups. **(D)** SCNA profiles with gains (red) and losses (blue) between the lower-risk and higher-risk groups. **(E)** Differential analysis of the fraction (%) of the genome altered, lost, and gained between the lower-risk and higher-risk groups. P values were obtained by the Mann-Whitney test. The asterisks represented the statistical P-value (*P < 0.05).

Genomic data, including mutation profile and somatic copy number alteration (SCNA) data from the TCGA-STAD dataset, were first analyzed to explore the possible mechanisms underlying the RNA processing-related prognostic signature. A significantly higher tumor mutation burden (TMB) was detected in the low-risk group than in the high-risk group (**Figure 6A**). More mutations caused more neoantigens in cases with lower risk scores (first vs. fourth quartile; **Figure 6B**) (45). After filtering out genes with low-frequency mutations (5% of GC samples), we found 25 significantly mutated genes between the two groups (**Figure 6C**). All these significantly mutated genes were enriched in the low-risk group, and were involved in the UV response down pathway (adjusted p = 0.014). Subsequently, investigation of the data related to SCNA events revealed distinct chromosomal alteration patterns between the low-risk and high-risk groups (**Figure 6D**). A significantly greater fraction of genome gained was detected in the low-risk group (**Figure 6E**).

**Figure 7:**
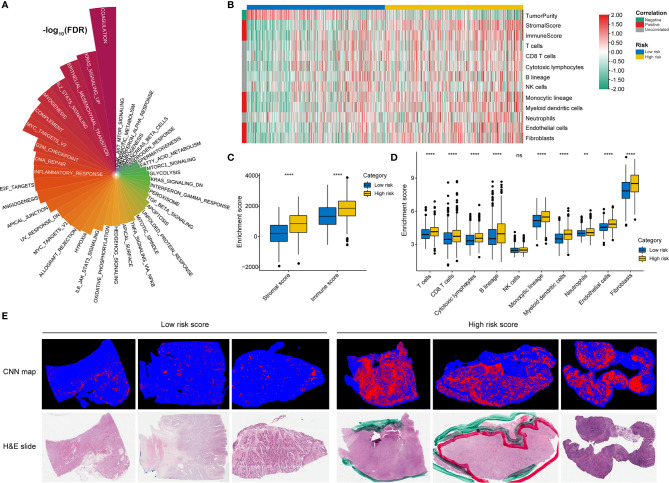
Identification of immune heterogeneity underlying the RNA processing-related prognostic signature. **(A)** The differential analysis of GSVA scores between the low-risk and high-risk groups. The height and color represent the -log10(FDR). **(B)** Heatmap showing the immune cell infiltration between the high-risk and low-risk groups. **(C)** Differential expression of immune scores and stromal scores between the low-risk and high-risk groups. P values were obtained by the Mann-Whitney test. The asterisks represented the statistical P-value (ns, not statistical; **P < 0.01; ****P < 0.001). **(D)** Differential expression of immune and stromal cells between the low-risk and high-risk groups. P values were obtained by the Mann-Whitney test. The asterisks represented the statistical P-value (ns, not statistical; **P < 0.01; ****P < 0.001). **(E)** Tumor-infiltrating lymphocyte infiltration between the low-risk and high-risk groups determined by hematoxylin–eosin (H&E) whole-slide images. Red represents a positive TIL patch; blue represents a tissue region with no TIL patch, while black represents no tissue.

GSVA confirmed significant differences in biological functions between the high-risk and low-risk groups (**Figure 7A**). Consistent with the above results, stromal activation pathways, such as the EMT, TGF-beta, and angiogenesis pathways, were significantly enriched in the high-risk group (**Supplementary Table 4**). The immune-related pathways, such as the complement, interferon-alpha response, and interferon-gamma response pathways, were also significantly enriched in the high-risk group (**Figure 7A** and **Supplementary Table 4**).

As the high-risk group had marked enrichment of the stromal and immune activation pathways, we explored the relationship between the tumor microenvironment status and the RNA processing-related signature to characterize their immune heterogeneity. We found that both the stromal and immune scores, representing stromal and immune cell infiltration in tumor tissue, respectively, were significantly higher in the high-risk group (**Figures 7B, C**). The MCP-counter algorithm also determined a higher proportion of immune and stromal cells in the high-risk group (**Figures 7B, D**). Further, based on the pathology whole-slide images, samples with high risk scores had a higher percentage of tumor-infiltrating lymphocytes (including T cells, B cells, and natural killer cells) than those with low risk scores (**Figure 7E**) (46). These results indicate that the activation of stromal and immune components in the tumor microenvironment and the activated oncogenic pathways based on the proposed signature likely contribute to the worse prognosis in high-risk patients.

### RNA Splicing Events Underlying the RNA Processing-Related Prognostic Signature

**Figure 8:**
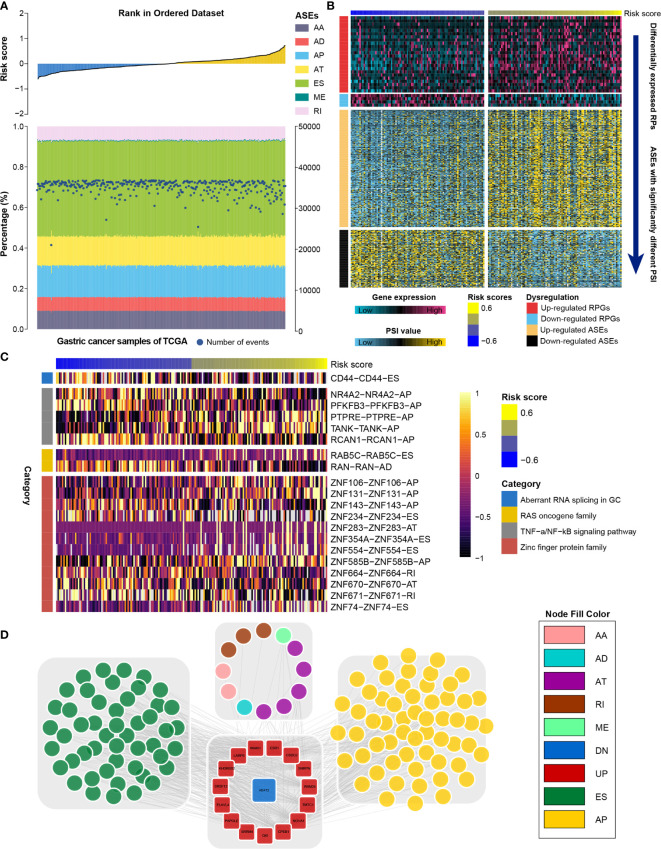
Identification of the RNA splicing landscape underlying the RNA processing-related prognostic signature. **(A)** Proportions of ASEs in 375 TCGA samples sorted by increased risk score. Bars indicate the proportion of each ASE type. Dark blue dots indicate the number of ASEs in each sample. The risk scores in ascending order are shown in the top panel. **(B)** Heatmaps showing the expression levels of RNA processing factors (top panel) and PSI value of ASEs with significant differences between lower-risk and higher-risk groups (bottom panel). **(C)** Representative ASEs with differential PSI values between lower-risk and higher-risk groups. **(D)** Network plot showing the correlation between RNA processing factors and ASEs with significant differences between lower-risk and higher-risk groups.

RNA processing factors dominate RNA splicing activities. Our outcomes showed that prognosis-associated RNA processing genes are closely correlated with RNA splicing-related activities (**Figure 8A**). Accordingly, we also comprehensively characterized ASEs in GC samples with different risk scores. Tens of thousands of seven ASE types were detected in each GC sample (**Figure 8A**). The proportion of these ASE types in the GC samples varied widely, from 0.5% to approximately 43% (**Figure 8A**). Although all GC samples shared similar ASE type patterns, the total number of detected ASEs gradually decreased along with the increasing risk score (p < 0.001, R = -0.18). Moreover, ASEs were significantly higher in GC samples with lower risk scores (first quartile, n = 94) compared to those with higher risk scores (fourth quartile, n = 94) (**Supplementary Figure 4**).

We further identified differentially expressed RNA processing genes (absolute fold change > 1.2, false discovery rate [FDR] < 0.05) and ASEs with significantly different PSI values (absolute fold change > 1.5, FDR < 0.05) in GC samples with lower risk (first quartile, n = 94) and higher risk (fourth quartile, n = 94) scores (**Figure 8B**). We identified 358 ASEs from 327 genes, including 240 upregulated ASEs from 217 genes and 118 downregulated ASEs from 118 genes (**Supplementary Table 5**). For these ASEs with markedly different PSI values, we found that the frequency of all ASE types was significantly altered compared to the background ASEs (**Supplementary Figure 5**), suggesting that the presence of altered ASEs might be associated with GC prognosis.

We found that genes involved in the aberrant RNA splicing in GC (*CD44*), the RAS oncogene family (*RAB5C*, *RANN*), the TNF-α/NF-κB signaling pathway (e.g., *NR4A2*, *TANK*, *PFKFB3*), and the zinc finger protein family (e.g., *ZNF74*, *ZNF671*, *ZNF106*) were differentially spliced among GC samples with lower and higher risk scores (**Figure 8C**). We performed GO analysis of all differentially spliced genes to explore the role of alternative splicing underlying the RNA processing-related signature. These spliced genes were mainly related to cell–matrix adhesion and mesenchymal cell differentiation for biological process; cell projection membrane and cell–substrate junction for cellular component; and cadherin binding and guanyl nucleotide exchange factor activity for molecular function (**Table S6**). Our analysis indicates that differential ASEs participate in many cancer-related pathways, suggesting that ASEs are a critical mechanism underlying the prognostic value of RNA processing factors in GC.

RNA splicing might inevitably affect their protein characteristics. Therefore, we constructed a protein interaction network based on the spliced genes, presenting the interactive relationship in normal conditions and uncovering the potential influence of ASEs at protein level. After removing the isolated nodes, 228 genes were mapped in the protein interaction network. These spliced genes were closely linked to each other (**Supplementary Figure 6**). From the whole protein interaction network, we identified six individual modules using the MCODE algorithm (47) (**Supplementary Figure 7**). Module enrichment analysis showed that most modules had biological functions with module specificity (**Supplementary Table 7**).

We explored the potential regulatory network among the significantly altered RNA processing genes and ASEs. A network with 549 pairwise correlations that ultimately involved 16 RNA processing genes and 119 ASEs was constructed (**Figure 8D**). Almost all ASEs followed the same expression trend as the RNA processing genes (**Supplementary Figure 8**). Most RNA processing genes were correlated with more than one ASE, and some played opposite roles in regulating different ASEs (**Figure 8D**). Besides, we found that different RNA processing genes competed for the same ASEs, partly explaining the diversity of splice isoforms created by only a few RNA processing factors.

### Drug Response Features Underlying the RNA Processing-Related Signature

**Figure 9:**
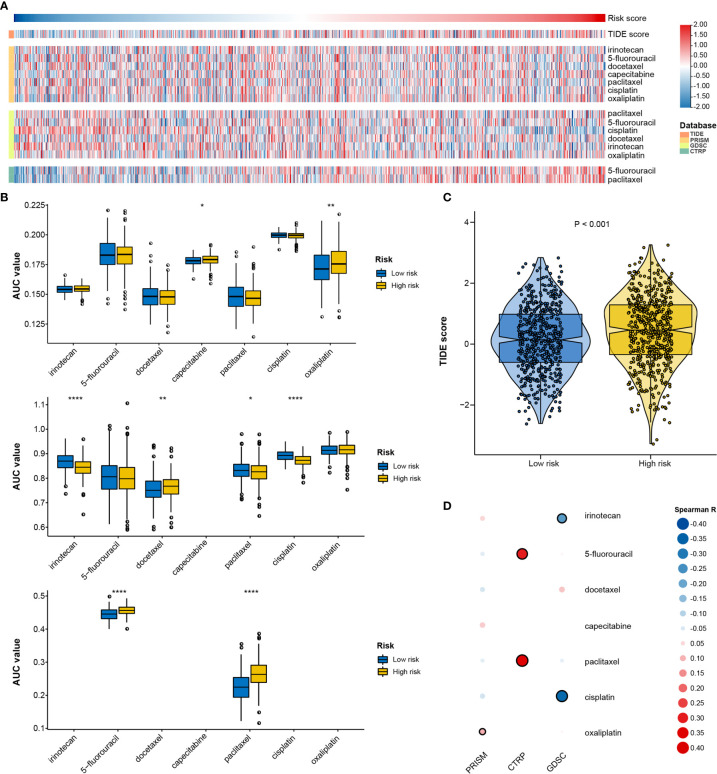
Identification of drug response features underlying the RNA processing-related signature. **(A)** Heatmap showing the TIDE scores and AUC values for patients with GC with different risk scores. **(B)** Differential analysis of the selected chemotherapeutic agents for patients with GC with higher and lower risk scores in the PRISM, CTRP, and GDSC databases. P values were obtained by the Mann-Whitney test. The asterisks represented the statistical P-value (*P < 0.05; **P < 0.01; ****P < 0.001). **(C)** Violin plots of TIDE scores for patients with GC in the high-risk and low-risk groups. **(D)** Heatmap showing the Spearman correlation coefficient between the AUC values and the risk scores for the selected chemotherapeutic agents.

Given that genetic variants, pathway activation, immune heterogeneity, and splicing features were significantly different according to the RNA processing-related signature, we investigated the relationship between the prognostic signature and drug response to encourage personalized treatment decisions. As described earlier, the low-risk group presented a significantly higher TMB and neoantigens count than the high-risk group (**Figures 6A, B**), suggesting that the patients with low risk scores might benefit from immune checkpoint inhibitor treatment. Consistent with the idea, the TIDE algorithm determined that patients with low risk scores (45.56%, 246/540) might be more likely to respond to immunotherapy than those with high risk scores (33.58%, 181/539) (p < 0.001, odds ratio [OR] = 1.654, 95% CI: 1.284–2.134) (**Figures 9A, C** and **Supplementary Figure 9**).

We used two approaches to identify the drug response relationship between the selected chemotherapeutic agents and the identified signature. The analyses were performed using GDSC, CTRP, and PRISM-derived drug response data. First, differential drug response analysis between the higher-risk (first quartile) and lower-risk (fourth quartile) groups was conducted to identify chemotherapeutic agents with significantly different AUC values (|mean difference| > 0.01, p < 0.05). Next, Pearson correlation analysis between the AUC value and the risk score was performed to select agents with a significant correlation coefficient (|R| > 0.1, p < 0.05). Finally, we determined that patients with low-risk scores were more sensitive to two CTRP-derived compounds (5-fluorouracil and paclitaxel), and patients with high-risk scores were more sensitive to two GDSC-derived compounds (irinotecan and cisplatin) (**Figures 9A, B, D**).

## Discussion

In this study, we found that the general expression pattern of RNA processing factors correlates with specific clinical outcomes and hallmark features of GC. RNA processing factors that were significantly associated with the prognosis of patients with GC were also identified. We then constructed a 10-gene RNA processing-related prognostic signature to predict the prognosis of stratified patients with GC. The identified signature was integrated with clinical features to establish the composite prognostic nomogram, which reliably demonstrated accurate prognostic predictions for the patients. Finally, we identified the clinical outcomes, genetic variants, pathway activation, immune heterogeneity, alternative splicing landscape, and drug response features associated with the prognostic signature.

GC is a highly heterogeneous malignant tumor. Some patients with GC within the same TNM stage have differing responses to treatment and prognosis (6). Therefore, further stratification of patients with GC with definite TNM subgroups is urgently needed. RNA plays a crucial role in cell biological functions by passing genetic information from DNA to protein and regulating various biological processes (48). Dysregulation of RNA profiles is closely related to the malignant progression and prognosis of GC. The RNA expression profile and RNA fate are highly dependent on the RNA processing factors responsible for precise temporal and spatial coordinating gene expression (49). Here, we highlight the stratification ability of RNA processing factors in GC.

In the present study, we identified five distinct RNA processing patterns, characterized by different biological behaviors and prognoses (**Figure 1**). We confirmed the prognostic value of a signature built with 10 RNA processing genes in each cohort (**Figure 2** and **Table 1**). The risk score of the RNA processing-related signature was a stable, independent prognosis factor in both the training and validation datasets (**Tables 2**, **3**). Moreover, we established a composite nomogram by integrating the RNA processing-related signature with traditional stratifying factors (age and TNM stage). The composite nomogram showed improved prognostic accuracy, better predictive efficiency, and larger net benefits than the signature alone and the prognostic model of the traditional stratifying factors in each cohort (**Figure 3**). These results indicate that the signature is a powerful tool for predicting the prognosis of patients with GC stratified by TNM classification.

The RNA processing-related signature reflects the expression alterations of genes involved in multiple vital hallmarks in GC. We found that genes that correlated negatively with the signature were significantly enriched in the pathways associated with proliferation and metabolism. In contrast, genes with expression that related positively to the signature’s risk score were significantly enriched in the invasion, metastasis, and immune biological processes (**Figure 4**). Among the 10 survival-related genes included in the signature, the risk-associated genes *PWP2* and *TGFB1* have been suggested to be associated with GC invasion and metastasis (50, 51), and *ZBTB7A*, a protection-associated gene, plays a tumor-suppressive role in GC cells (52). *METTL2B *was found to be RNA methyltransferases and play important roles in tumorigenesis (53). *CACTIN* involved in the regulation of innate immune response (54), contributing to the regulation of transcriptional activation of NF-kappa-B target genes in response to endogenous proinflammatory stimuli (55). *TRUB2* was a component of a functional protein-RNA module, which was required for intra-mitochondrial translation (56). *POLDIP3* was involved in regulation of translation, enhancing translational efficiency of spliced over non-spliced mRNAs (57). *TSEN54* participated the complex process for identification and cleavage of the splice sites in pre-tRNA. *SUGP1* and *RBMS1* were involved in RNA binding, playing a role in pre-mRNA splicing. These outcomes indicate that our study protocol can identify novel carcinogenesis-associated RNA processing genes that might serve as potential therapeutic targets. Future studies of these prognostic factors could identify novel mechanisms underlying RNA processing in GC.

We also determined that genetic variants, immune heterogeneity, and the alternative splicing landscape were also significantly different between the high-risk and low-risk groups. The low-risk group had significantly higher TMB, more neoantigens, and greater fraction of genome gained than the high-risk group (**Figure 6**). Consistent with the GSEA result, the stromal and immune activation pathways were markedly enriched with increased risk scores (**Figure 4**). The ESTIMATE and MCP-counter algorithms and the pathology whole-slide images also suggested a higher proportion of immune and stromal cells in the high-risk group (**Figure 7**).

Currently, genome-wide analyses have begun to reveal the roles of ASEs correlated with GC progression and prognosis (12, 13). Abnormal ASEs of individual genes participate in several tumorigenic processes, such as proliferation, apoptosis, hypoxia, angiogenesis, immune escape, and metastasis (58, 59). For example, *CD44* splice variants participate in GC carcinogenesis, progression, and metastasis (60, 61). By revealing the ASE landscape in GC, we identified 358 ASEs correlated with GC prognosis. We also observed that *CD44* was differentially spliced in the lower-risk and higher-risk groups (**Figure 8**). Moreover, we identified the potential regulatory network between the altered RNA processing genes and the differential ASEs.

Further, we investigated the relationship between the signature and drug response to promote personalized treatment decisions. To date, immune checkpoint inhibitors have been approved for GC treatment. However, the response rate is relatively low (10–26%) (62–64). Therefore, it is critical to find new biomarkers for appropriate patient selection for immunotherapy. We determined that patients with low risk scores might benefit from immune checkpoint inhibitor treatment (**Figure 9**), suggesting that this RNA processing-related signature could be a predictive biomarker for immunotherapy in GC.

Chemotherapy remains the mainstay in GC treatment (43). We found that patients with low-risk scores might be more sensitive to 5-fluorouracil and paclitaxel, both cell cycle-nonspecific drugs. 5-Fluorouracil is an anti-cancer antimetabolite that inhibits tumor cell proliferation *via* DNA damage. Paclitaxel stabilizes microtubules and interferes with mitotic spindle formation, which leads to the inhibition of cancer cell proliferation. As mentioned above, the proliferation- and metabolism-related pathways were markedly enriched with decreased risk scores. The activation of these pathways, such as G2M checkpoint, DNA repair, and mitotic spindle, might be responsible for the higher sensitivity to 5-fluorouracil and paclitaxel.

On the other hand, patients with high-risk scores might be more sensitive to irinotecan and cisplatin, cell cycle-nonspecific anti-cancer drugs. Such drugs are not affected by the cell cycle phase and act upon rapidly dividing cancer cells for destruction. Therefore, GC characterized with a mesenchymal phenotype might be more sensitive to irinotecan and cisplatin. Whether a genetic variant, pathway activation, immune heterogeneity, splicing features, or chemotherapy and immunochemotherapy response feature, all the results aid understanding of the roles of RNA processing in GC. Our signature may further aid the design of a more reasonable and effective treatment regimen, contributing to precision therapy for individual patients with different risk levels.

This study has several strengths. First, we analyzed a large sample of 1079 patients with GC using either RNA-seq or microarray data, suggesting that our outcomes are likely highly reliable, robust, and independent of specific expression quantitative platforms. Second, the present study includes both our own RNA-seq dataset and public datasets, indicating the possibility of future verification of our risk signature in additional cohorts. Third, we used RMS time to demonstrate the clinical utility of the RNA processing-related signature. It is equivalent to the area under the Kaplan-Meier curve from the beginning of the study through that time point. The RMST difference means gain or loss in the event-free survival time between the groups during this period. As such, using the average survival time can be more easily understood by clinical communities. Meanwhile, RMST difference is valid and interpretable whether or not the proportional hazards assumption is violated (65). Despite these strengths, our study has its limitations as well. First, we used only two clinical characteristics (age and TNM stage) to construct the composite nomogram; additional clinical factors, such as Lauren subtype, microsatellite instability status, chemotherapy, surgery, and radiotherapy information, are warranted to refine the model. Second, further *ex vivo*, *in vitro*, and *in vivo* experiments regarding these prognosis-related RNA processing factors are required to validate our *in silico* results. Finally, the response of immunotherapy and chemotherapy should be further verified by clinical data in other cohorts.

In summary, our study highlights the prognostic value of RNA processing genes in GC and reveal an RNA processing-related prognostic signature for further improving the prognosis prediction of patients with GC with definite TNM subgroups. The clinical outcomes, genetic variants, pathway activation, immune heterogeneity, splicing features, and drug response features underlying the signature were also identified. Our findings provide a basis for understanding the roles of RNA processing and indicate the potential clinical implications of RNA processing factors in GC.

## Data Availability Statement

The datasets presented in this study can be found in online repositories. The names of the repository/repositories and accession number(s) can be found in the article/**Supplementary Material**.

## Ethics Statement

The studies involving human participants were reviewed and approved by Institutional Review Board of the Harbin Medical University Cancer Hospital. The patients/participants provided their written informed consent to participate in this study.

## Author Contributions

SL, FM, and YX contributed to conception and design of the study. SL and FM organized the database. SL performed the statistical analysis. SL wrote the first draft of the manuscript. SL, FM, XY, YZ and BH wrote sections of the manuscript. All authors contributed to the article and approved the submitted version.

## Funding

This work was supported by funding from the Project Nn10 of Harbin Medical University Cancer Hospital (Grant Number Nn102017–03).

## Conflict of Interest

The authors declare that the research was conducted in the absence of any commercial or financial relationships that could be construed as a potential conflict of interest.

## Publisher’s Note

All claims expressed in this article are solely those of the authors and do not necessarily represent those of their affiliated organizations, or those of the publisher, the editors and the reviewers. Any product that may be evaluated in this article, or claim that may be made by its manufacturer, is not guaranteed or endorsed by the publisher.

## Supplementary Material

Supplementary Figure 1 The workflow of this study.

Supplementary Figure 2 Identification of the soft threshold according to the standard of the scale-free network. The red line represents the threshold line of 0.85.

Supplementary Figure 3 Intra-modular analysis for the signature-related modules. The scatterplot showing gene significance vs. module membership in the turquoise **(A)**, yellow **(B)**, and brown **(C)** modules.

Supplementary Figure 4 The absolute numbers of all ASEs were compared in GC patients with higher-risk (first quartile) and lower-risk (fourth quartile) scores.

Supplementary Figure 5 The distribution of splicing types for the differential and background ASEs. **(A)** The histogram showing ASE types’ frequency for the differential and background ASEs. **(B)** The pie graph showing ASE types’ frequency for the differential ASEs. **(C)** The pie graph showing ASE types’ frequency for the background ASEs.

Supplementary Figure 6 The protein interaction network for the spliced genes with significantly different PSI values.

Supplementary Figure 7 The six individual modules from the protein interaction network determined by the “MCODE” algorithm.

Supplementary Figure 8 The number of ASEs regulated by the 16 RNA processing factors.

Supplementary Figure 9 Response rates of immunotherapy between two risk groups determined by the “TIDE” algorithm.

Supplementary Table 1 929 genes, annotated as RNA processing factors, acquired in the AmiGO database.

Supplementary Table 2 819 RNA processing factors involved in all data sets.

Supplementary Table 3 105 OS associated RNA processing factors.

Supplementary Table 4 Difference of GSVA scores between the high-risk and low-risk groups.

Supplementary Table 5 358 differentially expressed ASEs and 28 differentially expressed RNA processing genes.

Supplementary Table 6 GO analysis was performed for all differentially spliced genes.

Supplementary Table 7 Module enrichment analysis for the six individual modules.
