# Supplementary figures and images for: Comprehensive Characterization of RNA Processing Factors in Gastric Cancer Identifies a Prognostic Signature for Predicting Clinical Outcomes and Therapeutic Responses
Source: Front Immunol. 2021 Aug 3;12:719628. doi: 10.3389/fimmu.2021.719628 (PMC8369824; doi:10.3389/fimmu.2021.719628)

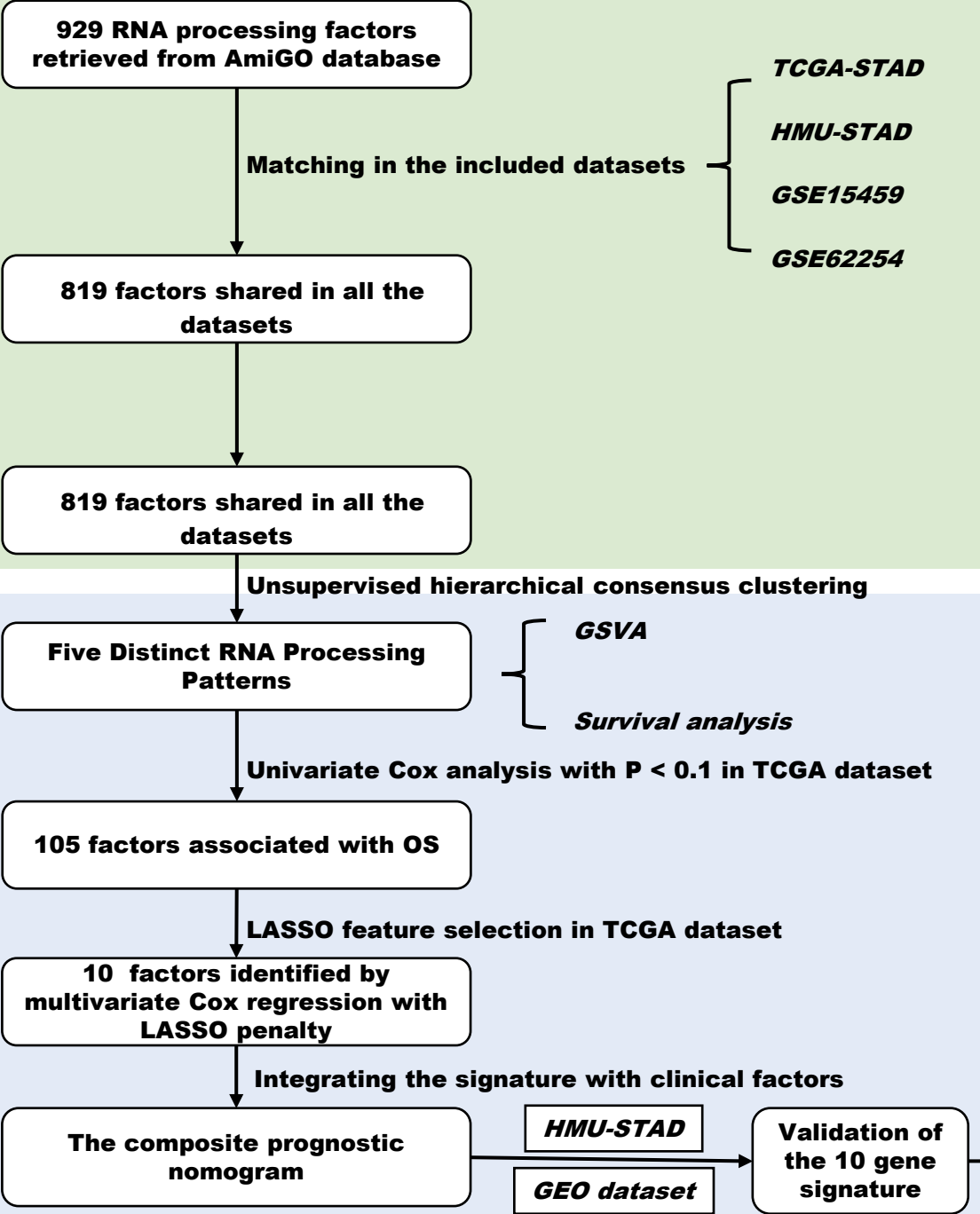

**Multi-omics features underlying the signature**

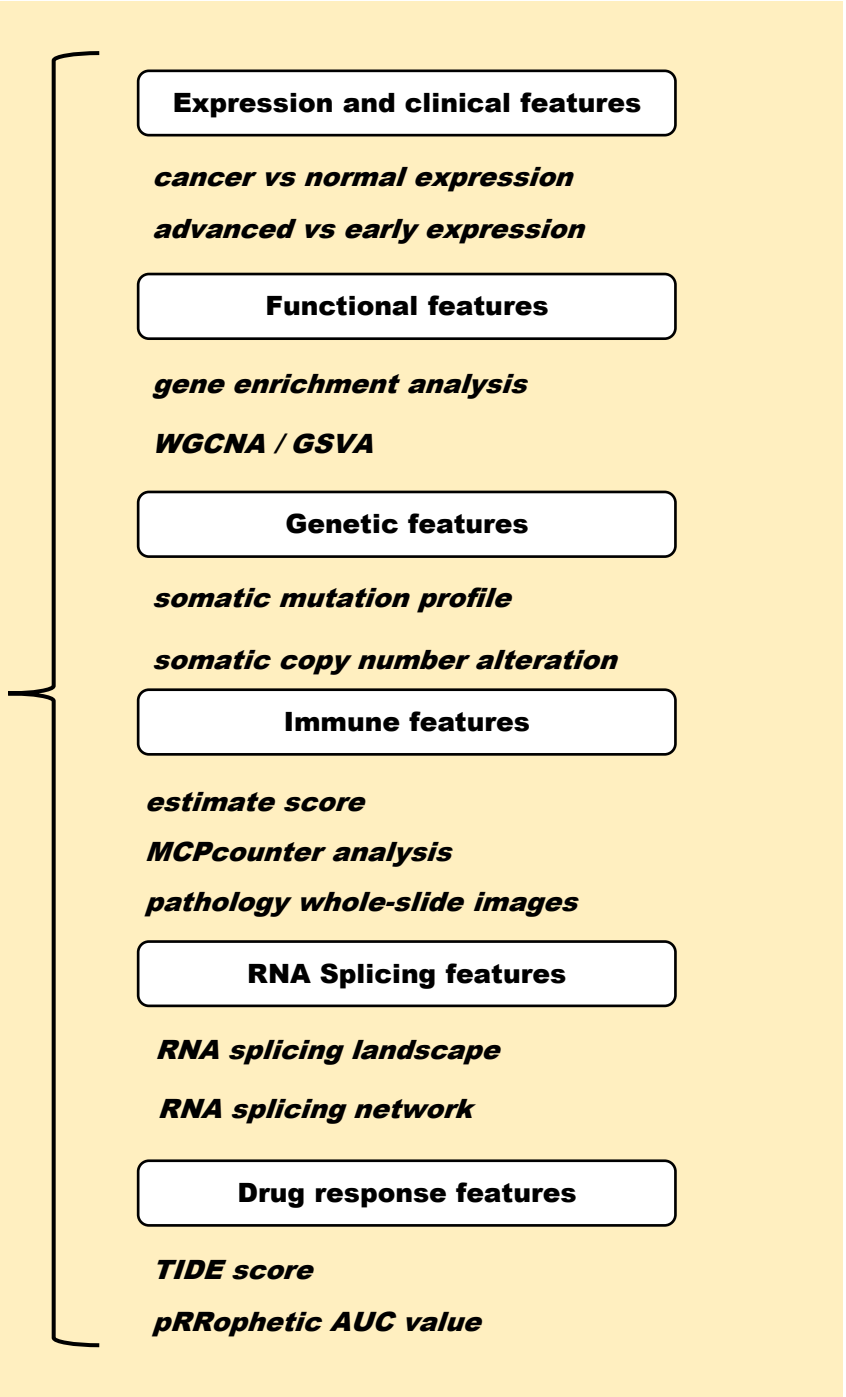

Supplement: Supplementary Figure 1 — The workflow of this study. [file DataSheet_1.pdf]

### Scale independence

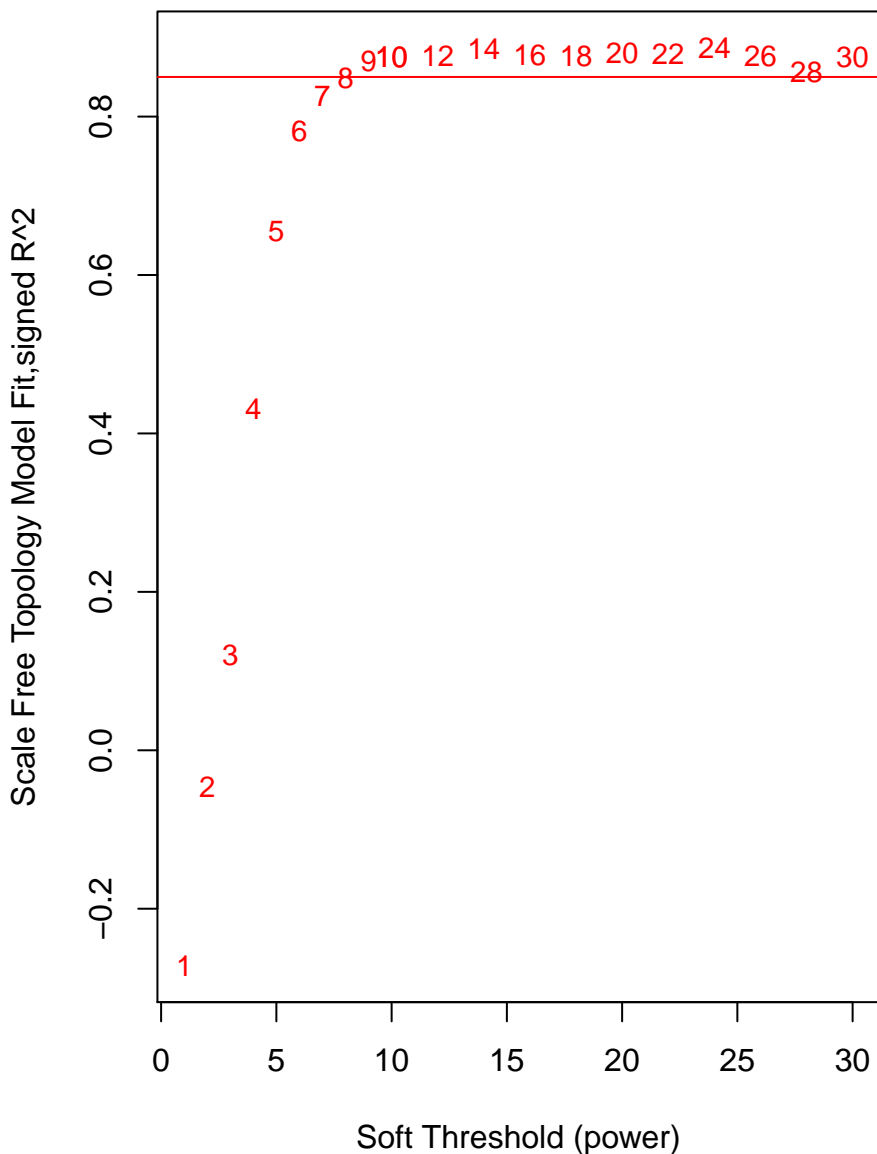

### Mean connectivity

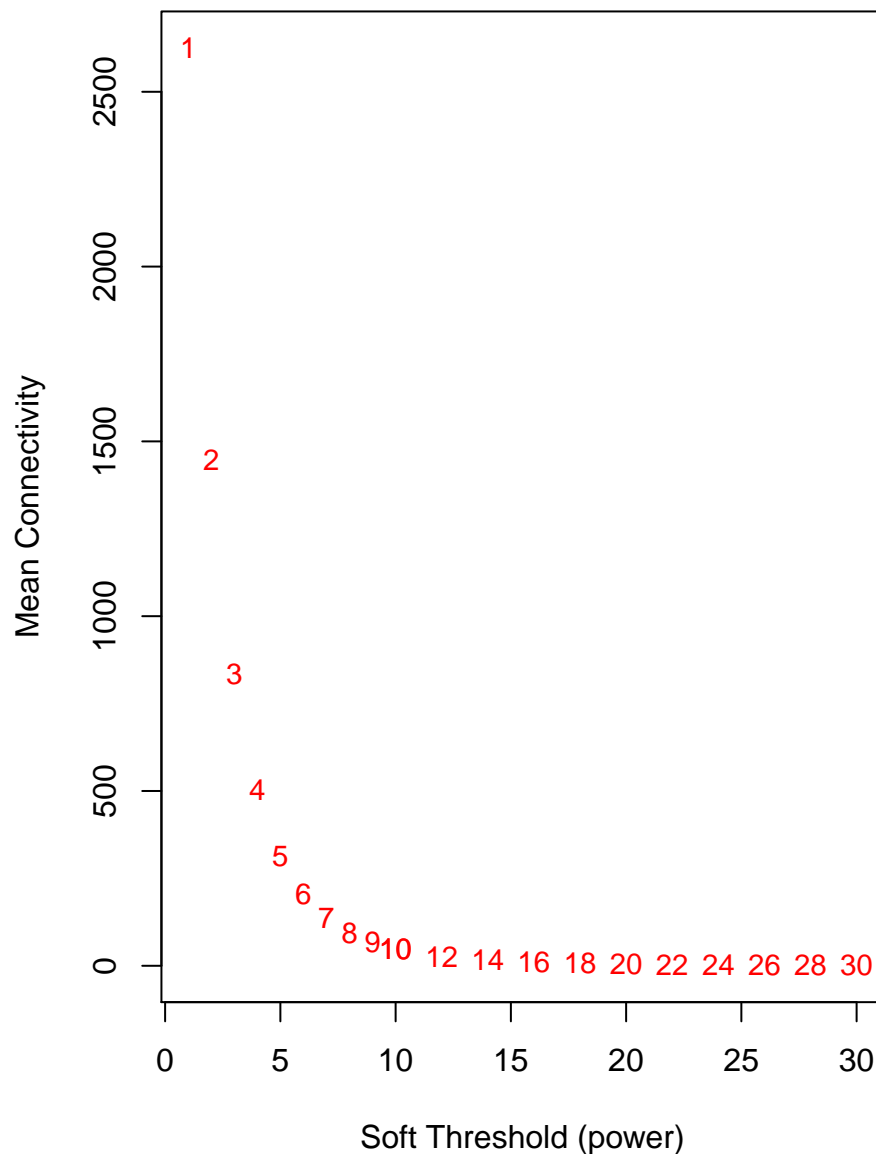

Supplement: Supplementary Figure 2 — Identification of the soft threshold according to the standard of the scale-free network. The red line represents the threshold line of 0.85. [file DataSheet_2.pdf]

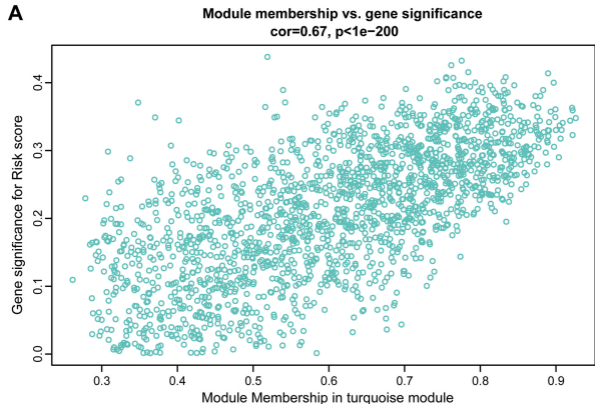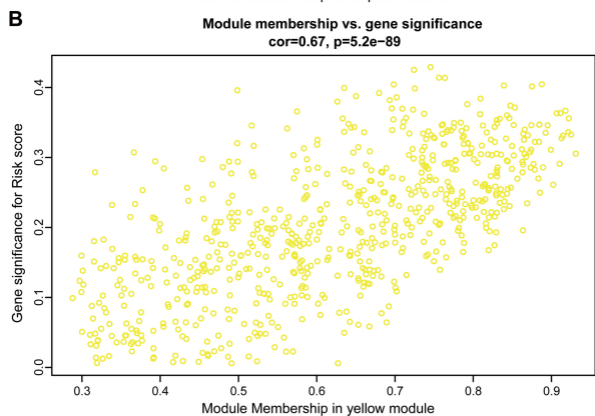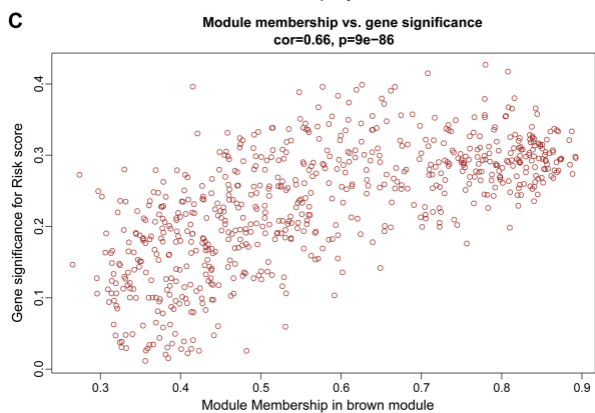

Supplement: Supplementary Figure 3 — Intra-modular analysis for the signature-related modules. The scatterplot showing gene significance vs. module membership in the turquoise (A), yellow (B), and brown (C) modules. [file DataSheet_3.pdf]

ASE counts

35000

30000

25000

Wilcoxon,  $p = 0.011$

LRisk

HRisk

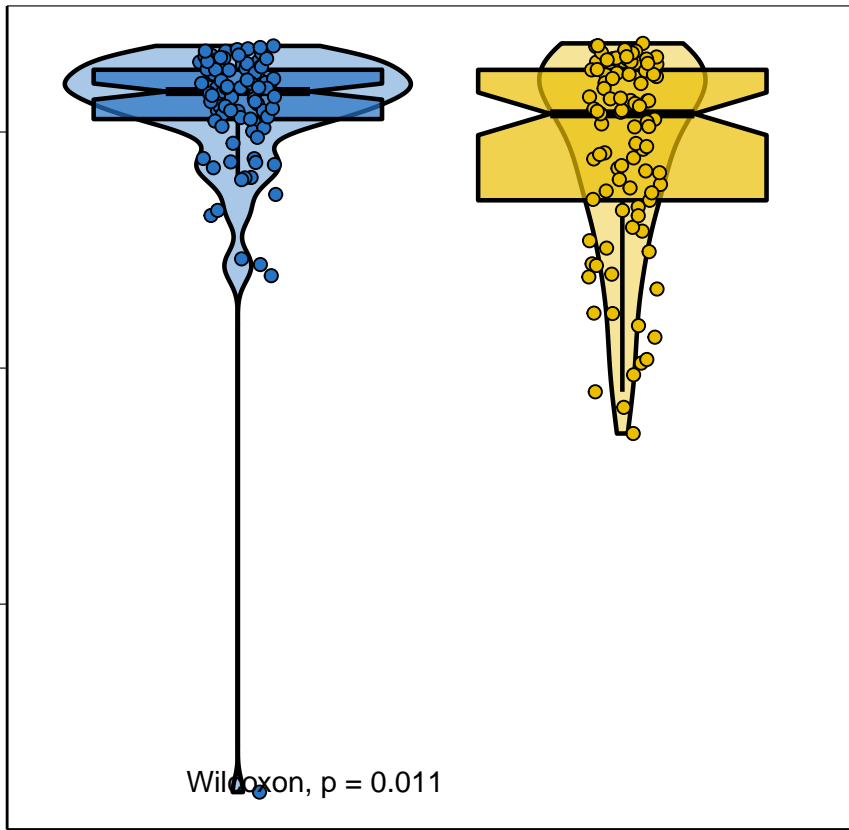

Supplement: Supplementary Figure 4 — The absolute numbers of all ASEs were compared in GC patients with higher-risk (first quartile) and lower-risk (fourth quartile) scores. [file DataSheet_4.pdf]

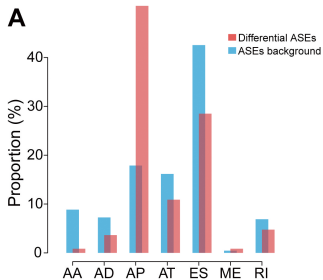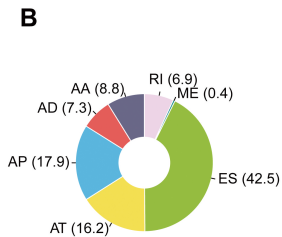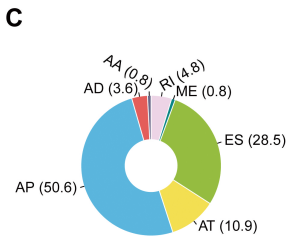

Supplement: Supplementary Figure 5 — The distribution of splicing types for the differential and background ASEs. (A) The histogram showing ASE types’ frequency for the differential and background ASEs. (B) The pie graph showing ASE types’ frequency for the differential ASEs. (C) The pie graph showing ASE types’ frequency for the background ASEs. [file DataSheet_5.pdf]

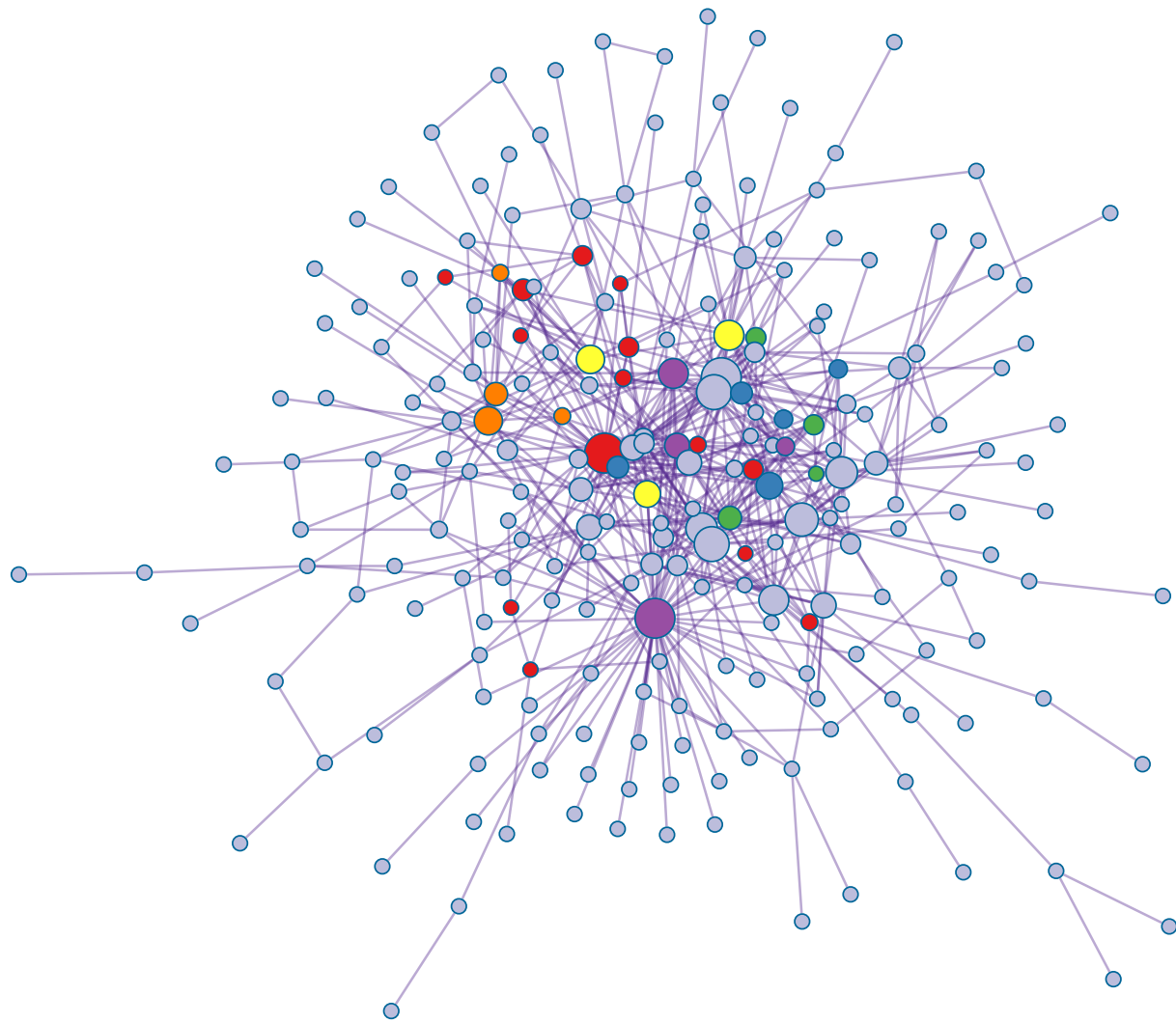

Supplement: Supplementary Figure 6 — The protein interaction network for the spliced genes with significantly different PSI values. [file DataSheet_6.pdf]

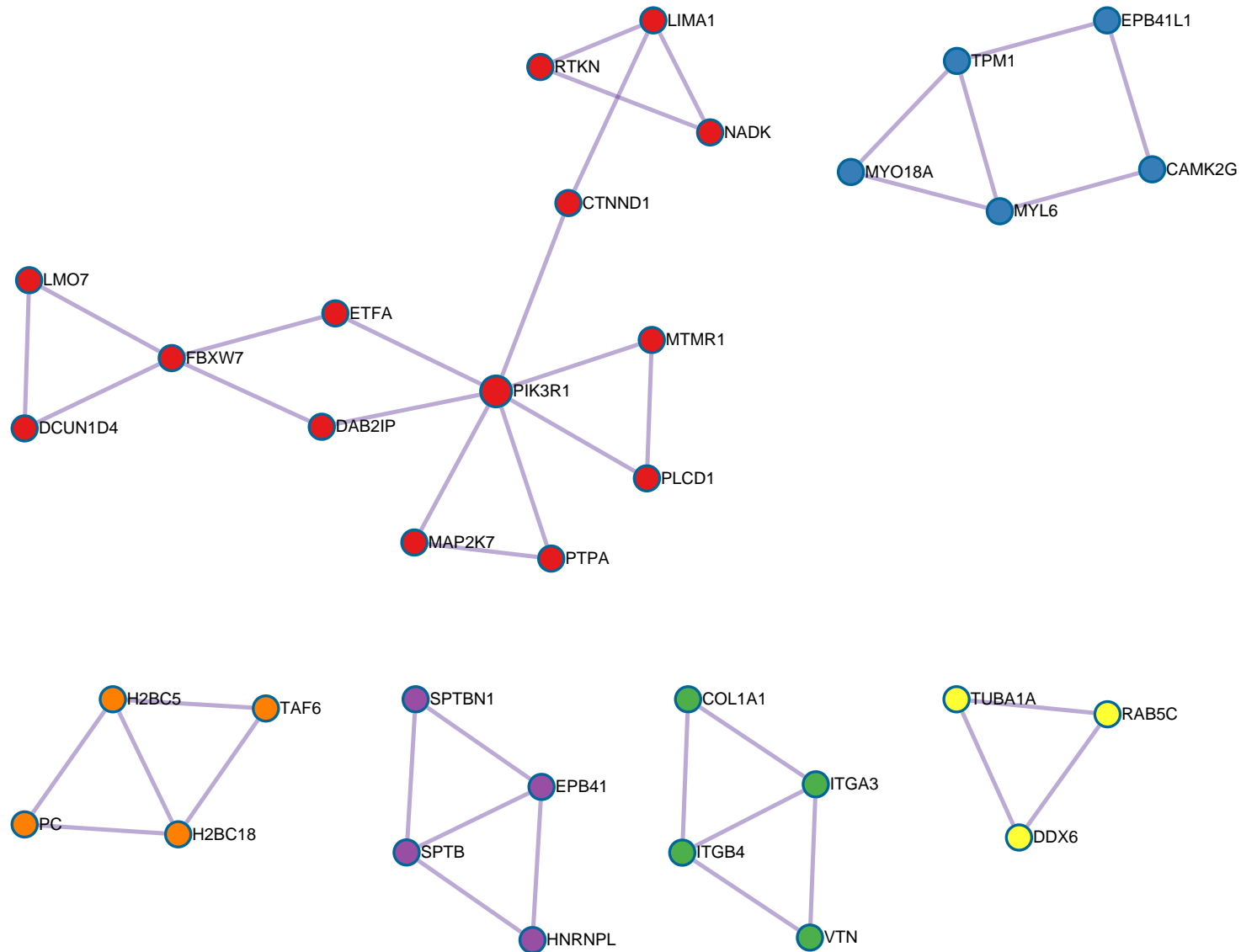

Supplement: Supplementary Figure 7 — The six individual modules from the protein interaction network determined by the “MCODE” algorithm. [file DataSheet_7.pdf]

Up

Down

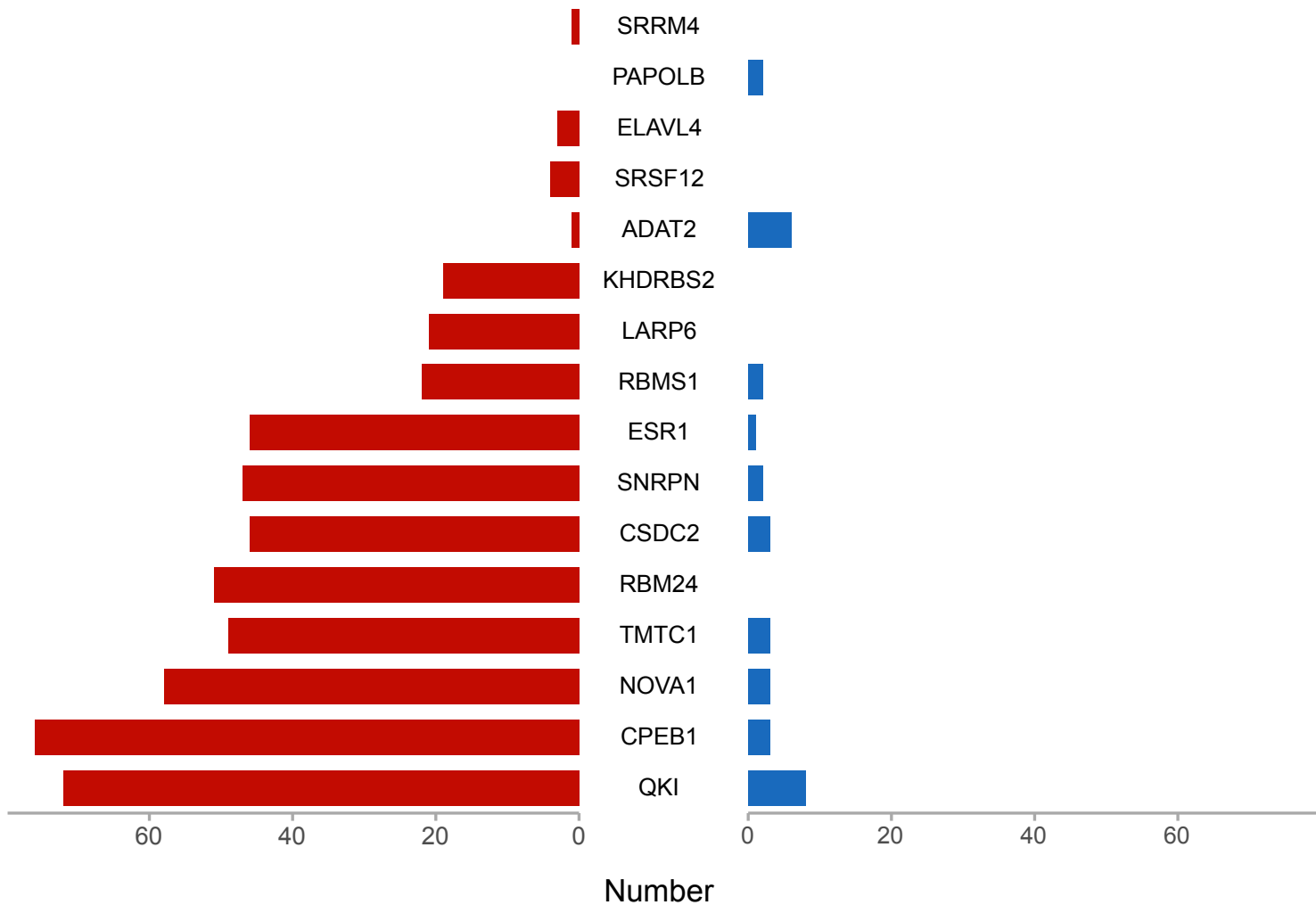

Supplement: Supplementary Figure 8 — The number of ASEs regulated by the 16 RNA processing factors. [file DataSheet_8.pdf]

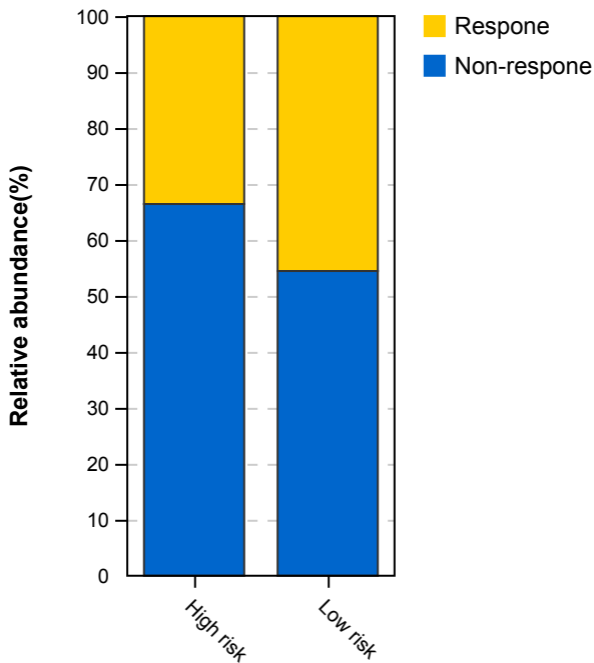

Supplement: Supplementary Figure 9 — Response rates of immunotherapy between two risk groups determined by the “TIDE” algorithm. [file DataSheet_9.pdf]
